# From diverticulum to diagnosis: The distinctive role of Endoscopic Ultrasonography in Lemmel syndrome

**DOI:** 10.1371/journal.pone.0327855

**Published:** 2025-07-09

**Authors:** Xin Chen, Xingkang He, Xiaoli Chen, Qian Cao

**Affiliations:** Department of Gastroenterology, Zhejiang University School of Medicine Sir Run Run Shaw Hospital, Hangzhou, Zhejiang, China; Hokkaido University: Hokkaido Daigaku, JAPAN

## Abstract

**Background and aim:**

Lemmel syndrome is a rare cause of obstructive jaundice, induced by periampullary diverticulum(PAD). Endoscopic Ultrasonography(EUS) is helpful in detecting PAD and differentiating the obstruction cause in Lemmel syndrome by exposing the detail of ampulla structure and measuring the dilatation of common bile duct and pancreatic duct.

**Materials and methods:**

Nineteen patients diagnosed Lemmel syndrome by EUS in our institute between January 1, 2019 to April 30, 2024 were enrolled. Their demographics, laboratory and imaging data were analyzed.

**Results:**

The patients diagnosed with Lemmel syndrome were mostly elder male with the average age of 68.8 ± 2.4 years. We found Lemmel syndrome patients most present with a dilatation in middle and upper segments of common bile duct. Magnetic resonance cholangiopancreatography (MRCP), abdominal computed tomography (CT) and upper abdominal enhanced MR were all failed to diagnose Lemmel syndrome. Besides, a higher serum level of alkaline phosphatase was predominant in those complicating with cholecystitis or cholecystolithiasis than those without complications.

**Conclusions:**

Similar with non-invasive imaging examination, EUS was capable of confirming the diagnosis of Lemmel syndrome, evaluating PAD and pancreaticobiliary duct as well as periampullary structure. EUS could be a useful investigate method to distinguish Lemmel syndrome for patients with unexplained dilated common bile duct or jaundice.

## Introduction

PAD is an abnormal sac adjacent to or containing the ampulla of Vater, which is formed by duodenal mucosa and muscularis mucosa. PAD is common and is usually asymptomatic. However, it can also induce some clinical conditions, such as hemorrhage, perforation, inflammation and pancreaticobiliary disorders. Pancreaticobiliary disorders caused by PAD include jaundice, choledochectasia, choledocholithiasis, cholangitis, pancreatitis and so on [1].

PAD compressing the distal common bile duct(CBD) induces obstructive jaundice without choledocholithiasis, tumor or other common causes of obstruction. This condition is recognized as Lemmel syndrome [2–4], first described by Lemmel in 1934 [5]. As a rare disease, there is few research about Lemmel syndrome. We investigated the clinical characteristics of Lemmel syndrome patients who were successfully evaluated by EUS, aim to establish the definite diagnosis.

## Materials and methods

From January 1, 2019 to April 30, 2024, a total of nineteen patients presented with jaundice and unknown cause of bile duct dilatation after different imaging studies, and we diagnosed them as Lemmel syndrome according EUS findings in our institution. All data were fully anonymize and accessed on May 1, 2024. Hematologic and biochemical tests including total bilirubin(TB), conjugated bilirubin(CB), alanine transaminase(ALT), aspartate transaminase(AST), alkaline phosphatase(ALP) and gamma glutamyl transpeptidase(GGT) were performed before EUS. All patients underwent enhanced abdominal CT and/or MRCP to exclude the detectable periampullary lesions. All continuous data are expressed as mean ± standard error of the mean (SEM) and analyzed using Student’s t-test to compare different groups separated by different complications. Fisher’s exact test was used for categorical data. *P* values < 0.05 were considered significant. The statistical analyses were performed anonymously with Excel for Windows(Microsoft, United States). This study received written ethics approval from Ethics Committee of Zhejiang University School of Medicine Sir Run Run Shaw Hospital (Acceptance No. 2023-735-01, Approval NO. 20230429). The informed consent could be exempted according to the ethics committee approval letter.

## Result

### Characteristics of patients

Nineteen patients were enrolled in this study, included fifteen males(78.9%) and four females(21.1%) with a mean age of 68.8 ± 2.4 years(range:50–89 years). All patients suffered from abdominal pain which is predominantly localized to the upper right quadrant, and three patients also accompanied with vomiting. The complications included acute pancreatitis(AP) (5/19, 26.3%), cholecystitis(11/19, 57.9%) and cholecystolithiasis(9/19, 47.4%). All patients underwent several blood tests and imaging examinations (Table 1).

**Table 1:** Clinical characteristics of Lemmel syndrome patients.

Age (year)	68.8 ± 2.4
Male sex (n)	15 (78.9%)
TB (μmol/L, normal range 0.0–26.0)	72.3 ± 14.0
CB (μmol/L, normal rang 0.0–4.0)	33.1 ± 8.44
ALT (U/L, normal range 9–50)	222.1 ± 31.5
AST (U/L, normal range 15–40)	226.1 ± 48.1
ALP (U/L, normal range 45–125)	190.4 ± 23.3
GGT (U/L, normal range 10–60)	468.7 ± 99.6
Imaging examinations (n)	
Abdominal enhanced CT	17
MRCP	16
Abdominal Routine CT	1
Upper-abdomen enhanced MR	2

TB = total bilirubin, CB = conjugated bilirubin, ALT = alanine transaminase, AST = aspartate transaminase, ALP = alkaline phosphatase, GGT = gamma glutamyl transpeptidase, CT = computed tomography, MRCP = magnetic resonance cholangiopancreatography, MR = magnetic resonance.

### Ultrasonic feature of EUS for Lemmel syndrome

**Fig 1:**
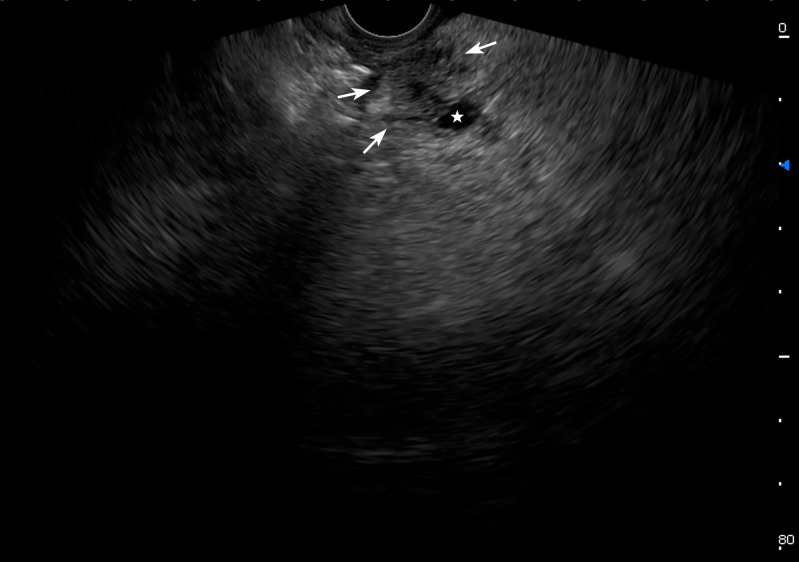
EUS image. EUS demonstrating a PAD at the the second portion of duodenal. Notice the CBD (white star) compression by the diverticulum (white arrowheads).

**Fig 2:**
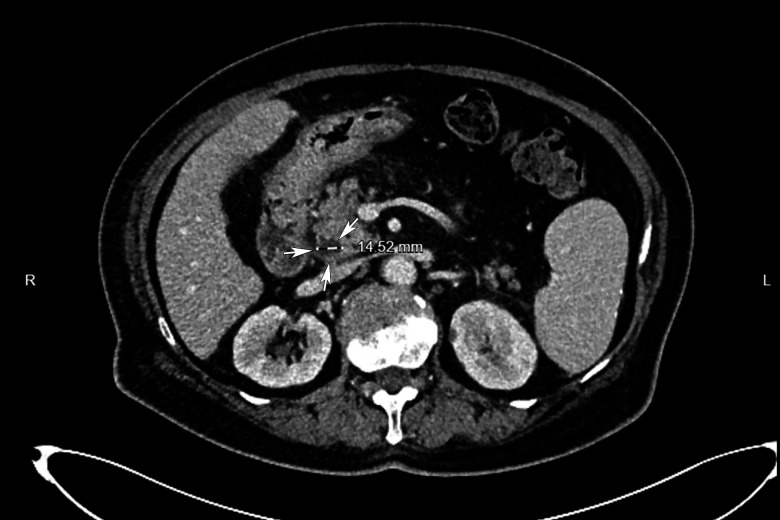
Enhanced abdominal CT scan. Coronal slice obtained through the pancreatic head and CBD. The CBD was dilated (white arrowheads) without PAD showing.

The diameter of pancreatic duct(PD) and CBD was measured with EUS. The average diameter of PD was 2.9 ± 0.2 mm, within normal range. A CBD diameter >7 mm was considered abnormal [6].This cutoff also applied to patients with prior cholecystectomy. In these patients, the average diameter of lower, middle, upper segments of CBD respectively were 6.0 ± 0.6 mm, 7.9 ± 0.6 mm and 8.7 ± 0.8 mm, revealing slight dilatation of the middle and upper segments of CBD. A Compression of CBD with PAD could be observed on the real-time scanning via EUS (Fig 1, S1 Video). A enhanced abdominal CT scan demonstrated diffuse exudation around the pancreatic head and adjacent duodenum without PAD in the same patient (Fig 2).

S1 Video. **EUS dynamic scan video.** EUS dynamic scan showing a PAD compressing the CBD.

### Imaging characteristics of CT and MR

In all nineteen patients, the PAD was revealed by imaging examinations in twelve patients, including eleven by enhanced abdominal CT, three by MRCP, two by upper abdominal enhanced MR, and one abdominal plain CT (Table 2).

**Table 2:** PAD detected by imaging examinations.

Total	12 (12/19, 63.2%)
Enhanced abdominal CT	11 (11/12, 91.7%)
MRCP	3 (3/12, 25.0%)
Upper abdominal enhanced MR	2 (2/12, 16.7%)
Abdominal plain CT	1 (1/12, 8.3%)

PAD = periampullary diverticulum, CT = computed tomography, MRCP = magnetic resonance cholangiopancreatography, MR = magnetic resonance.

We analyzed the characteristics in patients with imaging detectable PAD and those without. There was no significant difference between these two groups (Table 3).

**Table 3:** Differences of characteristics in PAD detected and non-detected group.

	PAD		
	Detected (n = 12)	Non-detected (n = 7)	*P* value
Age (year)	69.8 ± 2.7	67.0 ± 4.6	0.305
TB (μmol/L)	81.4 ± 20.6	56.8 ± 13.7	0.167
CB (μmol/L)	38.9 ± 12.3	23.2 ± 9.0	0.160
ALT (U/L)	204.8 ± 37.8	251.6 ± 57.9	0.257
AST (U/L)	217.9 ± 49.0	240.1 ± 106.1	0.427
ALP (U/L)	213.8 ± 33.4	150.4 ± 22.2	0.067
GGT (U/L)	503.1 ± 144.6	409.9 ± 118.9	0.312
CBD diameter (mm)			
Upper segment	8.1 ± 0.8	9.8 ± 1.6	0.179
Middle segment	7.9 ± 0.8	7.9 ± 1.2	0.500
Lower segment	6.0 ± 0.7	6.0 ± 0.9	0.484
PD diameter (mm)	2.7 ± 0.2	3.2 ± 0.5	0.178

PAD = periampullary diverticulum, TB = total bilirubin, CB = conjugated bilirubin, ALT = alanine transaminase, AST = aspartate transaminase, ALP = alkaline phosphatase, GGT = gamma glutamyl transpeptidase, CBD = common bile duct, PD = pancreatic duct.

### Complication of Lemmel syndrome patients

The Lemmel syndrome patient may get several complications, including AP, cholecystitis, cholecystolithiasis. In enrolled patients, five patients had gotten AP. Except for two patients who had already undergone cholecystectomy before, eleven patients suffered from cholecystitis, and nine patients had cholecystolithiasis. There was no significant difference in blood tests, the mean diameter of each segment of CBD, mean PD diameter and the sensitivity of CT/MRCP to detect PAD between the groups with AP compared with those without AP (Table 4). In the cholecystitis group, patients had a higher ALP level compared to the non-cholecystitis group (P = 0.015) (Table 5). The patients complicated with cholecystolithiasis also showed higher ALP than patients without cholecystolithiasis(P = 0.040) (Table 6).

**Table 4:** Differences of characteristics in patients with or without AP.

Factors	AP (n = 5)	Non-AP (n = 14)	*P* value
CBD diameter (mm)			
Upper segment	8.1 ± 1.6	8.9 ± 0.9	0.347
Middle segment	7.3 ± 1.4	8.2 ± 0.7	0.304
Lower segment	6.0 ± 1.3	6.0 ± 0.7	0.485
PD diameter (mm)	3.1 ± 0.5	2.8 ± 0.1	0.325
PAD revealed by CT/MR/MRCP	3 (60%)	9 (64%)	1.000
TB (μmol/L)	70.6 ± 18.8	73.0 ± 18.1	0.464
CB (μmol/L)	20.4 ± 3.2	37.7 ± 11.3	0.080
ALT (U/L)	234.0 ± 49.9	217.8 ± 39.8	0.402
AST (U/L)	339.8 ± 137.4	185.5 ± 42.4	0.167
ALP (U/L)	218.4 ± 35.5	180.4 ± 29.2	0.214
GGT (U/L)	518.0 ± 100.5	451.1 ± 132.1	0.346

AP = acute pancreatitis, CBD = common bile duct, PD = pancreatic duct, PAD = periampullary diverticulum, CT = computed tomography, MR = magnetic resonance, MRCP = magnetic resonance cholangiopancreatography, TB = total bilirubin, CB = conjugated bilirubin, ALT = alanine transaminase, AST = aspartate transaminase, ALP = alkaline phosphatase, GGT = gamma glutamyl transpeptidase.

**Table 5:** Differences of characteristics in patients with or without cholecystitis.

Factors	Cholecystitis (n = 11)	Non-cholecystitis (n = 6)	*P* value
CBD diameter (mm)			
Upper segment	7.7 ± 0.7	10.3 ± 2.0	0.131
Middle segment	7.36 ± 0.7	8.7 ± 1.4	0.218
Lower segment	5.2 ± 0.7	6.8 ± 0.9	0.091
PD diameter (mm)	2.9 ± 0.3	3.1 ± 0.5	0.353
PAD revealed by CT/MRCP	8 (73%)	3 (50%)	0.600
TB (μmol/L)	80.3 ± 20.2	66.03 ± 25.3	0.335
CB (μmol/L)	36.2 ± 11.7	33.6 ± 16.8	0.452
ALT (U/L)	243.3 ± 43.0	178.8 ± 62.6	0.208
AST (U/L)	238.3 ± 46.1	191.5 ± 125.3	0.369
ALP (U/L)	216.7 ± 35.7	123.8 ± 13.6	0.015*
GGT (U/L)	508.7 ± 155.5	335.3 ± 118.4	0.195

CBD = common bile duct, PD = pancreatic duct, PAD = periampullary diverticulum, CT = computed tomography, MR = magnetic resonance, MRCP = magnetic resonance cholangiopancreatography, TB = total bilirubin, CB = conjugated bilirubin, ALT = alanine transaminase, AST = aspartate transaminase, ALP = alkaline phosphatase, GGT = gamma glutamyl transpeptidase.

*P values < 0.05 were considered significant.

**Table 6:** Differences of characteristics in patients with or without cholecystolithiasis.

Factors	Cholecystolithiasis (n = 9)	Non-cholecystolithiasis (n = 8)	*P* value
CBD diameter (mm)			
Upper segment	7.7 ± 1.0	9.8 ± 1.4	0.120
Middle segment	7.1 ± 0.9	8.6 ± 1.0	0.153
Lower segment	5.3 ± 0.9	6.3 ± 0.7	0.181
PD diameter (mm)	2.8 ± 0.3	3.1 ± 0.4	0.252
PAD revealed by CT/MR/MRCP	7 (78%)	4(50%)	0.335
TB (μmol/L)	98.5 ± 21.9	49.1 ± 18.9	0.054
CB (μmol/L)	47.2 ± 13.4	21.9 ± 12.0	0.090
ALT (U/L)	237.1 ± 50.4	201.9 ± 51.6	0.316
AST (U/L)	247.6 ± 58.7	192.8 ± 89.9	0.309
ALP (U/L)	225.7 ± 42.1	137.0 ± 18.0	0.040*
GGT (U/L)	552.3 ± 177.6	329.6 ± 113.0	0.155

CBD = common bile duct, PD = pancreatic duct, PAD = periampullary diverticulum, CT = computed tomography, MR = magnetic resonance, MRCP = magnetic resonance cholangiopancreatography, TB = total bilirubin, CB = conjugated bilirubin, ALT = alanine transaminase, AST = aspartate transaminase, ALP = alkaline phosphatase, GGT = gamma glutamyl transpeptidase.

*P values < 0.05 were considered significant.

## Discussion

Lemmel syndrome is an obstructive jaundice induced by PAD compressing the distal CBD. Primary duodenal diverticulum are mostly solitary (90%) with no gender preference [7], and about 75% occur in the descending part of duodenum [8,9]. PAD appears in a 2–3 cm radius of the ampulla, but not containing it [10,11]. The incidence of PAD increases with age, reaching over 30% in patients aged more than 70 years [10–12]. Because PAD is the anatomical basis of Lemmel syndrome, we found that the mean age of Lemmel syndrome patients in our study was over 68 years, similar to that of the PAD patients in previous studies we mentioned before.

In some case reports and a recent literature review, patients with Lemmel syndrome may manifest with upper right quadrant abdominal pain, jaundice, nausea, vomiting, and fever [9,13,14]. Similarly, all patients suffered from abdominal pain and jaundice in our study. The clinical symptoms of Lemmel syndrome were non-specific and mainly consist of recurrent episodes of acute abdominal pain and jaundice, but also included cases with other symptoms. In clinical work, when receiving patients with unexplained jaundice accompanied by abdominal pain, Lemmel syndrome needs to be vigilant.

Lemmel syndrome could cause pancreaticobiliary complications including AP, acute cholecystitis and cholecystolithiasis by extrinsic compression of pancreaticobiliary duct by PAD [15]. In our study, cholecystitis was the most common complication in Lemmel syndrome patients, followed by cholecystolithiasis and AP. To investigate the potential clinical implications, we studied the relationships between age, lab test results, imaging features and the incidence of complications. Patients with Lemmel syndrome complicating cholecystitis or cholecystolithiasis had higher ALP. It may reveal a more severe obstruction in CBD which cause the complications and serve as a potential complication indicator. Therefore, Lemmel syndrome and PAD should be pay special attention in patients with biliary obstruction without evidence of stones, malignancy or infection. Blood tests including ALP may help us to discover Lemmel syndrome and be alarm to its complications.

Ultrasonography and other imaging examinations are significant to identify and diagnose Lemmel syndrome. PAD can be detected through EUS, enhanced CT scan, MRCP, ERCP and so on. Lemmel syndrome is usually confirmed by endoscopic examination [1,13,16,17]. ERCP is the gold standard for diagnosis and can be performed along with endoscopic intervention [1]. However, there have been increasing efforts to develop non-invasive and safer diagnostic modalities to assess the biliary tract due to its serious adverse effects such as post-ERCP pancreatitis [18]. In CT and MRI, PAD could appear as thin-walled cavitating lesions that located at the level of the 2nd part of the duodenum, arising from its medial wall. Unfortunately, abdominal CT scans lack specificity and a duodenal diverticulum can be misinterpreted as a possible pancreatic neoplasm or pseudocyst [19,20]. Case reports of Lemmel syndrome diagnosed by EUS are limited. EUS have good diagnostic efficiency on periampullary diseases [10,17,21]. CT, MRCP or other imaging examination could be inaccurate when PAD is compressed [10]. However, EUS has capability to provide real-time scanning under water, which can hold PAD up. We routinely recommend to employ EUS for the patients presenting with obstructive jaundice or dilation of the CBD/PD due to undetermined causes, especially the patients who has already undergone multiple imaging examinations yet remain difficult to diagnose. In our study, not all other imaging examinations can provides good visualization of PAD and diagnosis Lemmel syndrome. To the imaging examination inconclusive patients, combining with EUS may contribute to avoiding missed diagnosis of Lemmel syndrome.

Because Lemmel syndrome is a relatively rare condition, few studies existed with large sample sizes of Lemmel syndrome patients. Our research collected data from a certain amount of Lemmel syndrome patients, analyzed their demographic, clinical and imaging features. For exploring the future clinical application of EUS in Lemmel syndrome, we still faced several challenges and limitations. As a retrospective study, our entire sample population was limited to Lemmel syndrome patients diagnosed via EUS and sample size was small, which caused several statistical deficiencies. Our study was unable to definitively demonstrate the statistical superiority of EUS compared to other imaging examinations. We intend to conduct a prospective study focused on the clinical benefits of EUS. By expanding patient sample size, combining with appropriate data corrections for multiple comparisons, we hope to demonstrate the diagnostic value of EUS in patients with Lemmel syndrome more properly. The EUS should possess the potential to be an important instrument in the assessment of pancreaticobiliary diseases.

## Conclusion

Lemmel syndrome is a rare cause of obstructive jaundice. Patients are usually elderly, and most likely male. They may accompany with pancreaticobiliary complications, particularly when exhibiting elevated serum ALP levels. Compared with non-invasive imaging examination, EUS can evaluate PAD and pancreaticobiliary duct system dynamically and may be a useful investigate method to diagnose Lemmel syndrome from patients with unexplained dilated CBD or jaundice.

## Supporting information

S1 Video EUS dynamic scan video EUS dynamic scan showing a PAD compressing the CBD. (MP4)

S2 Data set. (XLS)
